# Cladribine-Based Therapy for Acute Myeloid Leukemia in Child, Adolescent, and Early Young Adult Patients: The MD Anderson Cancer Center Experience

**DOI:** 10.3390/cancers16223886

**Published:** 2024-11-20

**Authors:** David McCall, Shaikha Alqahtani, Moriah Budak, Irtiza Sheikh, Aaron E. Fan, Ramya Ramakrishnan, Cesar Nunez, Michael Roth, Miriam B. Garcia, Amber Gibson, Naval Daver, Sofia Garces, Nicholas J. Short, Ghayas C. Issa, Farhad Ravandi, Courtney D. DiNardo, Guillermo Montalban Bravo, Guillermo Garcia-Manero, Branko Cuglievan, Tapan Kadia

**Affiliations:** 1 Department of Pediatrics, The University of Texas MD Anderson Cancer Center, Houston, TX 77030, USA; 2 Department of Pediatric Stem Cell Transplantation and Cellular Therapy, The University of Texas MD Anderson Cancer Center, Houston, TX 77030, USA; 3 Department of Leukemia, The University of Texas MD Anderson Cancer Center, Houston, TX 77030, USA; 4 Department of Pathology, The University of Texas MD Anderson Cancer Center, Houston, TX 77030, USA

**Keywords:** acute myeloid leukemia, cladribine, purine analog, leukemia, pediatrics, adolescents

## Abstract

**Background:** Cladribine-based combination chemotherapy has demonstrated promising efficacy in patients with relapsed/refractory adult acute myeloid leukemia (AML), prompting its increased utilization in the frontline; in pediatrics, it has been typically reserved for relapsed or refractory cases. While fludarabine has been used more commonly as a purine analog in intensive regimens, cladribine may be an important alternative. **Methods:** We performed a retrospective study at MD Anderson Cancer Center from January 2015 to July 2023, which included patients aged 1–21 years with refractory or relapsed AML who received cladribine outside of a transplant conditioning. **Results:** A total of 30 patients were included, with a median age of 20 years (range, 2–21), and 55% being male. Similar to adults, cladribine exhibited good tolerability in pediatric and adolescent patients, with the most common adverse events being febrile neutropenia and myelosuppression. The most common grade 3 or 4 adverse events included febrile neutropenia (55%) and sepsis (26%), and there were no treatment discontinuations due to adverse events. Among patients with a median number of 2 (0–7) prior treatments, the overall response rate (CR/CRi) was 45%, and median event-free and overall survival were 6 and 12 months, respectively. Disease progression resulted in 4 deaths within 30 days of treatment. **Conclusions:** Cladribine was tolerated in pediatrics. No new safety signals were seen with cladribine regimens in this cohort. Response assessment is limited due to the heavily pretreated cohort. Further prospective studies are warranted on the safety and efficacy of cladribine and establish its role in pediatric, adolescent, and early young adult patients with AML.

## Simple Summary

Cladribine is a chemotherapy used in combination with other chemotherapy drugs in adult patients with acute myeloid leukemia (AML) who relapse or are nonresponsive to treatment. It appeared to work well and is now used for newly diagnosed adult patients. While it is commonly used in adults, it has not been used routinely in children. We looked at medical records of pediatric and adolescent patients treated for AML at MD Anderson to analyze its safety and how well it works against AML. It was found to be safe and well tolerated in these patients and also showed improved survival outcomes. About half of the patients responded to cladribine either by itself or with other chemotherapy agents. This review also highlighted its safety in combination with other agents. We found these combinations to be well tolerated and could provide additional options for pediatric providers who are treating relapsed AML.

## 1. Introduction

The 5-year event-free survival rates for childhood acute myeloid leukemia (AML) range between 49% and 64% [1,2], with relapse occurring in up to one-third of cases [3]. The long-term outcome of this subset of patients continues to be poor, with an overall survival rate under 40% [4]. To continue making progress against this aggressive childhood leukemia, novel drugs and novel combinations that overcome resistance and target distinct pathways are needed.

Cladribine is a synthetic purine nucleoside analog that is FDA-approved to treat symptomatic hairy cell leukemia [5]. Its design resists degradation by adenosine deaminase; cladribine intercalates into DNA and interferes with DNA synthesis in replicating cells, inhibits DNA repair, inhibits RNA synthesis, and has pro-apoptotic effects [6,7,8]. Clinically, cladribine has shown efficacy as a single agent in various hematologic malignancies, including not only hairy cell leukemia but also chronic lymphocytic leukemia and AML [6,9,10].

Cladribine has been used in combination with several regimens, including cytarabine, topotecan, idarubicin, and venetoclax [11,12,13,14,15,16]. In all of these studies, hematologic toxicities were the most common adverse events. While cladribine has been traditionally considered a salvage regimen for pediatric and adult patients, recent trends in the treatment of adult leukemia have evaluated cladribine in the frontline setting, yielding a 1-year survival rate of 78% [17].

Given the limited current data for this agent in the pediatric, adolescent, and early young adult patient population using newer AML combinations, we retrospectively reviewed our institutional experience at MD Anderson Cancer Center for patients with relapsed or refractory AML who received cladribine as salvage chemotherapy.

## 2. Materials and Methods

In accordance with Institutional Review Board approval, a retrospective chart review was conducted for patients who had undergone one or more cycles of cladribine-based (received more than one dose of cladribine) treatment for AML at our institution, excluding those who had received cladribine as part of conditioning regimens for HSCT. The study period spanned from January 2015 to July 2023. Inclusion criteria encompassed patients aged 21 years or younger who had a pathologic diagnosis of AML by World Health Organization (WHO) specifications [18]. Data for analysis encompassed patient demographics, treatment regimens, relapse history, mortality, and peripheral blood laboratory values (neutrophil count, hemoglobin, platelet count). The dosage, regimen, and number of cladribine cycles administered were documented. Complex karyotype was defined as more than or equal to 3 chromosomal aberrations. Bone marrow evaluations included assessment of mutational profile, cytogenetic analyses (categorized as normal/diploid, complex karyotype, or displaying other unique chromosome abnormalities) with or without fluorescence in situ hybridization, flow cytometry assessment, and molecular analysis via next-generation sequencing [19,20]. Response assessment was conducted following the Revised Recommendations of the International Working Group Response Criteria [21]. Flow cytometry assessment followed established protocols, while molecular analysis utilized a targeted next-generation sequencing panel focusing on genes frequently mutated in myeloid and lymphoid malignancies [19,20].

Adverse events (AE) associated with cladribine administration were based on clinical documentation. The AEs were graded in accordance with the Common Terminology Criteria for Adverse Events version 5.0 [22]. Descriptive statistics were employed to report efficacy and toxicity data.

Statistical analysis involves summarizing continuous variables via mean, median, and range and categorical variables via percentages and frequencies. Kaplan–Meier estimates were utilized to assess unadjusted overall survival time distributions. Data were compared using the log-rank test. All analyses were conducted using GraphPad Prism version 9.0.

## 3. Results

### 3.1. Patient Characteristics

Thirty-one patients were included in our study cohort. Their baseline characteristics are detailed in Table 1.

The median age was 20 (range: 2–21) years, with 17 (55%) patients being male. The median number of therapies prior to cladribine was 2 (0–7), including 7 (23%) patients who had received an SCT prior to receiving a cladribine-based salvage regimen, and 9 (29%) had prior venetoclax-based therapy. There were 2 patients (6%) with therapy-related AML. 22 (71%) of patients had adverse risk classification [23], including 16 (52%) who had a complex karyotype. The most frequent mutations were *FLT3* (*n* = 10, including 6 internal tandem duplication [ITD] and 2 tyrosine kinase domain [TKD]), *KRAS* (*n* = 8), *WT1* (*n* = 7), *NRAS* (*n* = 7), *KMT2A* (*n* = 5), *NPM1* (*n* = 4), and *TP53* (*n* = 4). Germline testing was done in two patients, and one patient had a germline *RUNX1* mutation. In addition to the high-risk characteristics listed in Table 1, for the relapsed patients, the median time from diagnosis to relapse was 11 months. Individual patient and disease characteristics, treatment regimens, and outcomes are shown in Table 2.

### 3.2. Treatment

All patients received cladribine in combination with either multiagent chemotherapy or hypomethylating agents (Table 2) (Supplemental Table S1 for dosing regimens). The median treatment duration was 1 cycle (range 1–7). The median dosing for cladribine was 5 mg/m^2^ (range 5–10 mg/m^2^). The most commonly used regimen was CLIA plus venetoclax (*n* = 11), which is composed of cladribine, idarubicin, high-dose cytarabine, and venetoclax as previously described [17]. The next most common regimen was CLIA (*n* = 5). Several additional patients were treated with targeted therapies in combination with CLIA backbone: venetoclax and gilteritinib (*n* = 2); venetoclax and gemtuzumab (*n* = 4); and either midostaurin, gilteritinib, or quizartinib (*n* = 1 each). Cladribine with cytarabine (high and low dose) was another common backbone regimen that was used in combination: gemtuzumab (*n* = 3), venetoclax (*n* = 3).

### 3.3. Safety Profile and Adverse Events

The adverse events associated with the cladribine regimens, regardless of drug attribution, are listed in Table 3.

Hematologic adverse events were frequently observed. Common grade 3 or 4 adverse events included febrile neutropenia (61%), aspartate aminotransferase (AST)/alanine aminotransferase (ALT) elevation (13%), anemia (19%), and thrombocytopenia (26%). No patients had to discontinue the medication due to adverse events. Only 1 patient (patient 18) had a dose reduction of cladribine from 5 to 3 mg/m^2^. No grade 4 tumor lysis was reported.

Grade 3 or 4 sepsis with proven bacteremia developed in 29% (*n* = 10) of patients. Isolated organisms included *Streptococci viridans* (*n* = 4), *Conidiobolus* (*n* = 1), *Streptococcus mitis* (*n* = 1), *Rothia* spp. (*n* = 1), *Escherichia coli* (*n* = 1), and *Staphylococcus aureus* (*n* = 1). One patient had a lung infection. Four patients (12.9%) died within 30 days of cladribine-based induction due to disease progression; there were no deaths attributed to infections.

### 3.4. Response

**Figure 1:**
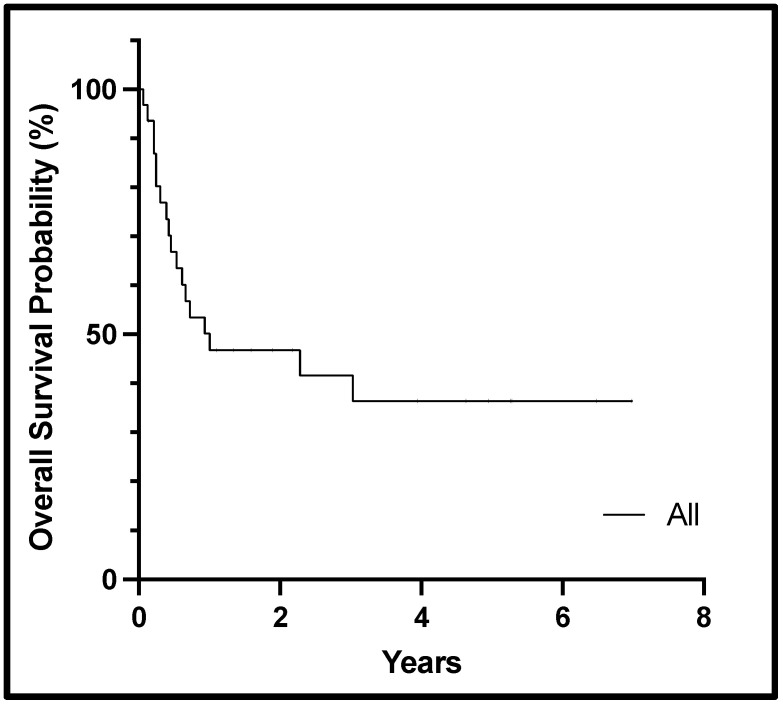
Kaplan–Meier curve showing overall survival from the start of cladribine therapy for the cohort.

Among the 31 patients, 14 (45%) had an overall response, including 3 (10%) CR and 11 (35%) Cri. Among those with CR/Cri, 6 (19%) had a complex karyotype, and the most frequent mutations included *FLT3* (*n* = 5), *NRAS* (*n* = 5), *NPM1* (*n* = 3), and *KRAS* (*n* = 3). Of the 14 responders, 8 (57%) patients went on to receive HSCT following a cladribine-based regimen: 3 utilizing haploidentical donors, 3 matched sibling donors, 1 matched unrelated donor, and 1 cord blood transplant. As seen in Table 2, several patients achieved remission and did not get consolidated with an HSCT. Reasons for not undergoing HSCT (*n* = 23, 74%) included failed pre-HSCT work-up (*n* = 2), MRD positivity (*n* = 2), patient preference (*n* = 1), unavailable donor and need for bridging chemotherapy (*n* = 1), and refractive/progressive disease (*n* = 17). Of the 17 non-responders, 14 (82%) had complex karyotypes, and 3 had *MECOM* rearrangement. Median event-free and overall survival from the time of cladribine dosing was 6 (range 1–63 months) and 12 months (range 1–84 months), respectively. Figure 1 displays the overall survival for all patients.

## 4. Discussion

While currently approved for use in patients with hairy cell leukemia, there has been an increased recognition of the potent activity of cladribine, particularly in combination with cytarabine, in the treatment of AML. This has generated increased utilization of cladribine within AML induction programs for adults with AML, both in the frontline and relapsed settings, compelling further exploration in the pediatric setting.

Santana et al. first reported the use of cladribine in pediatric patients with relapsed or refractory leukemia in 1991 [24]. Their phase 1 study revealed that cladribine, as a single agent, demonstrated greater activity in AML rather than in acute lymphocytic leukemia (ALL), and the dose-limiting effect was myelosuppression. Among 18 patients (aged 2–21 years), the complete response (CR) rate was 11%. A subsequent phase 2 study evaluated cladribine at a dose of 8.9 mg/m^2^/day for 5 days as monotherapy, producing a CR rate of 47%, with 7 of the 10 responders receiving hematopoietic stem cell transplantation (HSCT) [25]. Rubnitz et al. published their experience with cladribine in the St. Jude AML97 trial, in which 96 children (aged 0.05–21 years) were randomized to receive cladribine with one of two different dosing arms of cytarabine [26]. This trial utilized 5 days of cladribine at 9 mg/m^2^/dose and yielded a 5-year event-free survival and overall survival estimates of 44.1% and 50.0%. Grade 3–4 infection rates ranged from 26% to 38% after each course of chemotherapy. Ruan et al. published a retrospective analysis of children who received CLAG-M (cladribine, cytarabine, granulocyte colony-stimulating factor [G-CSF], and mitoxantrone) compared to MEC/IEC (mitoxantrone, etoposide, and cytarabine, or idarubicin, etoposide, and cytarabine) in pediatric patients (aged 1–15 years) with relapsed/refractory AML [27]. The CLAG-M group showed a remarkable overall response rate of 80% (16/20), compared to 51% (18/35) in the MEC/IEC group (*p* < 0.001). Here again, the most common grade 3–4 adverse events were hematologic toxicities. These studies are detailed in Supplemental Table S2.

In this single-institution report of cladribine use in patients aged 21 years or younger with AML, we found that cladribine was safe, well tolerated, and effective when used as part of salvage chemotherapy combinations. As commonly seen in this setting, febrile neutropenia and bloodstream infections were among the most common adverse events identified but were manageable. Additionally, while reporting small numbers, our series demonstrated novel combinations of cladribine (i.e., cladribine, low-dose cytarabine, and venetoclax; or cladribine with FLT3 inhibitors) [15,28] that were effective, with tolerable toxicity, that can be prospectively evaluated in a pediatric population.

Notably, in our study, cladribine-based salvage therapy was implemented in a cohort of patients who, in addition to having resistant disease, were heavily pretreated and had pre-existing organ dysfunction and complex karyotypes. Despite this, none of the patients required treatment discontinuation other than for the progression of the disease. Common grade 3 and 4 adverse events were mostly hematologic, and prophylactic antimicrobials and G-CSF were used. The toxicity profile recognized in this cohort was analogous to that found in the pivotal CLIA-venetoclax study of patients with AML and myelodysplastic syndrome (NCT02115295) [17], as well as in pediatric studies and reviews [26,27] where the most common grade 3–4 adverse events were hematologic toxicities. These studies included heavily pretreated AML patients with neutropenia and high blast percentage, and many of them were already hospitalized for infectious processes prior to initiating therapy.

Assessing the objective response to cladribine was not a primary goal in this retrospective review, as our patient population was limited in number and the combinations of concurrent therapy with cladribine were varied. Therefore, no reliable conclusions on efficacy can be made. Of the limited numbers, the regimen of CLIA/Venetoclax + GO or CLIA/Venetoclax yielded the most responses, with the caveat that CLIA/Venetoclax + GO may have excessive toxicities. This cohort’s overall response rate with or without blood count recovery was 45%, with a median overall survival of 12 months from the first cladribine dose. This was below the response rate for the AAML1421 study of children (aged 1.81–21.5 years) with relapsed AML, which yielded an overall response rate of 82% [29], and also the cladribine experience of Ruan et al. (80%) [27] and Rubnitz et al. (49–76%) [26]. However, a direct comparison cannot be made given our heterogeneous group experience (untreated patients versus several lines of therapy in this cohort and different drug combinations). Also of note, even though the response rate was not improved, this cohort was heavily pretreated.

One particular subset of interest is patients with *KMT2A* rearrangement (*KMT2A*-r). Preclinical data has shown that *KMT2A*-r cells have sensitivity to nucleoside analogs like cladribine [30]. However, patients with *KMT2A*-r have differing outcomes based on their partner gene [31]. In the last seven years, only two studies/case series have specifically commented on the effect of cladribine on this subset: Li et al. enrolled 30 patients, with 6 of those having *KMT2A*-r, with the following results: 1 CRi, 2 morphologic leukemia-free states, 1 partial response, and 2 with no response to the cladribine, cytarabine, or venetoclax regimen [32]. Ruan et al. retrospectively looked at 70 children with 1 *KMT2A*-r patient achieving CR after cladribine and cytarabine [27]. In this current study, there were 5 patients with *KMT2A*-r. While this subset is not powered to delineate strong conclusions, the patients that received multi-modal cladribine and cytarabine regimens with gemtuzumab or venetoclax did reach remission and ultimately stem cell transplant.

Given its clear activity, incorporating cladribine into frontline therapies needs to be studied in the pediatric population. Adult studies are currently using cladribine for frontline and relapsed populations with such combinations as cladribine plus low-dose cytarabine (LDAC) and venetoclax alternating with decitabine or azacitidine (NCT01515527, NCT05365035), sorafenib (NCT02728050), uproleselan (NCT04848974), imatinib (NCT00258271), gemtuzumab (NCT04050280), venetoclax-navitoclax (NCT06007911), BL22 immunotoxin (NCT00074048), bryostatin 1 (NCT00003174), or lintuzumab–Ac-225 (NCT03441048). Another study is testing the use of cladribine as an oral tablet (NCT04178005), which would ease administration and perhaps decrease hospital administration. Bataller et al. [33] reported on the phase II study of cladribine low-dose cytarabine and venetoclax in newly diagnosed patients (aged ≥60 years), observing a CR/CRi rate of 85% with a lower-intensity regimen in an older, unfit population. For pediatric patients who are not suitable for intensive chemotherapy, such as patients with Down syndrome, this could be a safe and effective option. For those who can tolerate intensive chemotherapy, the question remains as to whether a fludarabine-based regimen such as FLAG-Ida-Venetoclax (97% ORR in newly diagnosed adults) [34] or CLIA-Venetoclax is superior (94% ORR in newly diagnosed adults) [17]. Tinajero et al. [35] retrospectively looked at these two regimens in adults, and the response rates were similar, albeit it was a small sample size. Noting the excellent response rate in adult newly diagnosed AML patients warrants the discussion of bringing cladribine to the front line for pediatric patients.

In the relapsed or refractory pediatric population, the goal is to minimize disease to a level that enables SCT. Based on the experience detailed in this cohort, CLIA-venetoclax should be considered in first-relapse or refractory pediatric AML patients.

We acknowledge some limitations of this study, including its relatively small population size and its retrospective nature. We also recognize only 4 out of 31 patients are under 10 years of age, highlighting this was an older pediatric cohort. In addition, we acknowledge that the treatment approach and supportive care offered in major academic centers in North America may not be reflective of real-world clinical practice, and there are differences in treatment approach included in this analysis.

## 5. Conclusions

This is one of the largest published case series reporting the use of cladribine combinations in the pediatric, adolescent, and early young adult population for AML. Cladribine appears to be safe and well tolerated even in newer combinations. Patients should be monitored closely for prolonged myelosuppression and febrile neutropenia. However, more studies are needed to establish the optimal dose (both with and without concurrent chemotherapy) and length of therapy in this population and to collect long-term safety and activity data in children and adolescents. In summary, this case series demonstrates that cladribine-based regimens should be considered as salvage chemotherapy in patients with relapsed or refractory AML and should be further studied in pediatric clinical trials.

## Figures and Tables

**Table 1:** Baseline characteristics for the patients included in the cohort.

Baseline Characteristics	No. of Patients (%) (*n* = 31)
Median Age in Years (Range)	20 years (2–21)
Age Group, No.	
2–10 years	4
11–17 years	6
18–21 years	21
Sex	
Female	14 (45%)
Male	17 (55%)
Race	
White	12 (39%)
Hispanic	7 (22%)
African American	3 (10%)
Asian	2 (7%)
Other/Not Reported	7 (22%)
Median Number of Prior Therapies	2 (0–7)
Received HSCT After Cladribine Regimen	
Yes	8 (26%)
No	23 (74%)
Karyotype *	
Complex karyotype	20 (65%)
Diploid	16 (52%)
Hyperdiploid	7 (23%)
Hypodiploid	5 (16%)
Monosomy 7	4 (13%)

Abbreviations: HSCT, hematopoietic stem cell transplantation. * This is a summary of karyotypes included in our cohort. Some patients had more than 1 cytogenetic change.

**Table 2:** Patient and disease characteristics, treatment regimens, responses, and adverse events.

Diagnosis	Age/ Gender	Cytogenetics/Molecular Mutations	Risk [23]	No. of Lines of Prior Therapy	Cladribine Regimen	No. of Cladribine Cycles	Best Cladribine Response	Grade 3 or 4 Adverse Events	HSCT Before/After Cladribine Regimen
Frontline									
AML	20 y/M	*KRAS*, *NRAS*, *FLT3*-TKD, *CBFB* Rearrangement	Favorable	0	CLAD/ARAC + GO	6	CRi	Thrombocytopenia Grade 4, Febrile Neutropenia Grade 3	No/No
AML	21 y/F	*DNMT3a*, *NPM1*, *TET2*	Favorable	2	CLIA/Venetoclax	5	CRi	Febrile Neutropenia Grade 3, Anemia Grade 3	No/Yes
APL	19 y/F	*ASXL2*, *BCOR*, *RAD21*	Intermediate	0	CLIA/Venetoclax	4	CR	ALT elevation Grade 3	No/No
AML	21 y/F	*NRAS*, *FLT3*	Intermediate	1	CLIA/Venetoclax	4	CRi	Febrile Neutropenia Grade 3	No/No
AML	21 y/F	*FLT3-ITD*, *KDM6A*, *NPM1*, *ETV6*	Intermediate	2	CLIA/Venetoclax, CLIA + Gilteritinib, CLIA + Quizartinib	7	CRi	Febrile Neutropenia Grade 3	No/No
AML	21 y/F	*NPM1*, *FLT3*-ITD	Intermediate	1	CLIA/Venetoclax + Gilteritinib	2	CR	Thrombocytopenia Grade 4, Febrile Neutropenia Grade 3	No/Yes
AML/Germ cell tumor	17 y/M	*TP53*	Adverse	0	CLIA/Venetoclax	2	CRi	Thrombocytopenia Grade 4, Anemia Grade 3, Febrile Neutropenia Grade 3	No/Yes
t-AML	21 y/M	*KMT2A* Rearrangement	Adverse	0	CLIA/Venetoclax	3	CRi	Thrombocytopenia Grade 4	No/Yes
AML	21 y/M	*KMT2A* Rearrangement, *MYC* Rearrangement, *TP53*, *NRAS*	Adverse	4	CLIA	1	NR	Hypofibrinogenemia Grade 3, Febrile Neutropenia Grade 3	No/No
Relapsed or Refractory									
Rel-AML	13 y/M	*CEBPA*	Favorable	4	CLAD/ARAC	1	NR	Anemia Grade 3, Febrile Neutropenia Grade 3	Yes/No
Rel-AML	21 y/F	*KRAS*	Intermediate	2	CLIA	1	NR	Anemia Grade 3, Bronchopulmonary Hemorrhage Grade 4, Febrile Neutropenia Grade 3	Yes/No
Refr-AML	21 y/F	*WT1*	Intermediate	1	CLIA	1	CRi	None	No/No
Rel-AML	2 y/F	*MECOM* Rearrangement, *CBFA2T3-GLIS2*	Adverse	3	CLAD/ARAC + Venetoclax	2	NR	None	Yes/No
Refr-AML	18 y/M	*MECOM* Rearrangement, *CALR*, *CBL*, *FLT3*-ITD, *FLT3*-D835, *PTPN11*, *STAT5A*, *WT1*	Adverse	5	CLIA + Midostaurin	2	NR	Hypokalemia Grade 3, Febrile Neutropenia Grade 4	No/No
Refr-AML	19 y/F	*MECOM* Rearrangement, *NRAS*, *FLT3*-ITD, *KDM6A*, *ASXL1*, *ASXL2*, *ETV6*, *GATA2*, *KRAS*, *WT1*, *TERT*	Adverse	4	CLIA/Venetoclax + Gilteritinib	1	NR	Thrombocytopenia Grade 4	No/No
Rel-AML	15 y/M	*FLT3*-ITD, *NUP98-NSD1*, *WT1*	Adverse	4	CLIA/Venetoclax + GO	1	NR	Febrile Neutropenia Grade 3, Hypokalemia Grade 3	Yes/No
Rel-AML	21 y/M	*FLT3*-ITD, *TET2*, *WT1*	Adverse	4	CLIA	1	NR	None	Yes/No
Rel-AML	17 y/F	*FLT3*-TKD, *WT1*, *KMT2A* Rearrangement, *PRPF40B*	Adverse	6	CLIA/Venetoclax + GO	1	CRi	Febrile Neutropenia Grade 3	No/Yes
Refr-AML	11 y/M	*KMT2A* Rearrangement, *KRAS*	Adverse	1	CLAD/ARAC	1	CRi	Thrombocytopenia Grade 4	No/No
Refr-AML	3 y/M	*KMT2A* rearrangement, *ASXL1*, *KRAS*, *BCL6* Rearrangement	Adverse	4	CLAD/LDAC + Venetoclax	3	NR	Anemia Grade 3	No/No
Rel-AML	4 y/F	*TP53*, *GATA1*, *JAK3*, *SH2B3*, *SMC3*, *SUZ12*	Adverse	4	CLAD/ARAC + GO	1	NR	AST Elevation Grade 3, Hypokalemia Grade 4	No/No
Refr-AML/Germ cell tumor	18 y/M	*TP53*, *KRAS*	Adverse	0	CLIA/Venetoclax	1	NR	Hyperbilirubinemia Grade 3, Febrile Neutropenia Grade 3	No/No
Rel-AML	21 y/F	*KRAS*, *PTPN11*, *IKZF1*, *RB1*	Adverse	2	CLIA/Venetoclax	1	NR	Cardiomyopathy Grade 3, Lung Infection Grade 4	Yes/No
Refr-AML t-AML	21 y/M	*KRAS*	Adverse	3	CLIA/Venetoclax + GO	1	CRi	ALT Elevation Grade 3, Febrile Neutropenia Grade 3	No/Yes
Rel-AML	21 y/M	*NRAS*, *IKZF1*, *TERT*, *WT1*	Adverse	4	CLIA/Venetoclax	3	NR	None	No/No
Refr-AML	21 y/F	*NRAS*	Adverse	1	CLIA	2	CR	Thrombocytopenia Grade 4, Febrile Neutropenia Grade 3	No/Yes
Rel-AML	19 y/F	*DNMT3A*	Adverse	7	CLIA/Venetoclax	1	NR	AST Elevation Grade 3, Febrile Neutropenia Grade 4	Yes/No
Rel-AML	14 y/M	*NPM1*	Adverse	1	CLAD/ARAC + GO	1	NR	None	No/No
Rel-AML	8 y/M	None	Adverse	2	CLAD/ARAC + Venetoclax	2	CRi	Thrombocytopenia Grade 3, Febrile Neutropenia Grade 3, Anemia Grade 3	No/Yes
Rel-AML	21 y/M	*CEBPA*, *WT1*, *KIT*, *NRAS*	Adverse	4	CLIA/Venetoclax	1	NR	Febrile Neutropenia Grade 3	No/No
Rel-AML	21 y/M	*CEBPA*, *KIT*, *STAT5A*, *RUNX1-RUNX1T1* Rearrangement	Adverse	4	CLIA/Venetoclax + GO	1	NR	Hypokalemia Grade 3, Febrile Neutropenia Grade 3	No/No

Abbreviations: AML, acute myeloid leukemia; APL, acute promyelocytic leukemia; HSCT, hematopoietic stem cell transplantation; CLAD, cladribine; CLIA, cladribine, idarubicin, cytarabine; LDAC, low-dose cytarabine; ARAC, cytarabine; GO, gemtuzumab ozogamicin; PEG, PEG-asparaginase; NR, no response; CR, complete response; CRi, CR without blood count recovery; AST, aspartate aminotransferase; ALT, alanine aminotransferase; Rel-AML, relapsed; AML Ref-AML, refractory AML; t-AML, treatment-related AML.

**Table 3:** Grade 3 or 4 adverse events related to cladribine per CTCAE v5.0.

Adverse Event	No. of Patients		
	Grade 3/4	Grade 3	Grade 4
Febrile Neutropenia	19	17	2
Thrombocytopenia	8	1	7
Anemia	6	6	0
Elevated ALT/AST	4	4	0
Elevated Bilirubin	1	1	0
Hypokalemia	4	3	1
Cardiomyopathy	1	1	0
Lung Infection	1	0	4
Bronchopulmonary Hemorrhage	1	0	1
Hypofibrinogenemia	1	1	0

Abbreviation: ALT/AST, alanine aminotransferase/aspartate aminotransaminase.

## Data Availability

The original data presented in this study are available on request from the corresponding author. The data are not publicly available due to confidential patient information.

## Supplementary Materials

The following supporting information can be downloaded at: https://www.mdpi.com/article/10.3390/cancers16223886/s1, Table S1: Treatment Details of Cladribine-Containing Regimens; Table S2: Cladribine Studies in Pediatric AML.
