# Supplementary material for: Cladribine-Based Therapy for Acute Myeloid Leukemia in Child, Adolescent, and Early Young Adult Patients: The MD Anderson Cancer Center Experience
Source: Cancers (Basel). 2024 Nov 20;16(22):3886. doi: 10.3390/cancers16223886 (PMC11592422; doi:10.3390/cancers16223886)
Supplement: Supplementary file 1 [file cancers-16-03886-s001.zip › Supplement Table S2_CLAD study table.pdf]

Supplemental Table 2: Cladribine Studies in Pediatric AML							
**Study**	**Number of Patients**	**Median Age (years)**	**Study Design**	**Cladribine Dose**	**Cladribine Regimen Details**	**Outcome**	**Common Adverse Events**
Santana et al (1992)	N=24	11 (3-19)	Phase II trial	8.9mg/m2/d	Continuous for 5 days	CR: 47%<br>PR: 12%	Grade ≥ 3 Neutropenia<br>Grade ≥ 3Thrombocytopenia
Rubnitz et al (2009)	N=96	9 (0.05-21)	Phase III trial	9 mg/m2/dose	Arm A: 5 days cladribine, cytarabine (500mg/m2/dose) over 2 hours.<br><br>Arm B: 5 days cladribine, cytarabine continuous infusion (500 mg/m2/d)	Arm A:43%<br>Arm B: 65%	Grade ≥ 3 toxicity more in Arm B (48%) compared to Arm A (24%)
Inaba et al (2010)	N=26	10 (1-19)	Phase I trial	8.9mg/m2/d	5 days cadribine and topotecan (4mg/m2/d)*	CR: 34.6%<br>PR : 61.5%	Febrile neutropenia
Chaleff et al (2012)	N=104	9.2 (0.9-20.4)	Phase II	8mg/m2/d	5 days cladribine<br>3 days idarubicin(10mg/m2/d)	CR: 46%<br>PR: 5%	Grade ≥ 3 Neutropenia<br>Grade ≥ 3Thrombocytopenia

Abbreviation: Complete remission (CR) , partial remission (PR) , day(d)
*Topotecan was individualized after day 1 to target systematic exposure of 140 ±20mg/ml.hr
