# Supplementary material for: Cladribine-Based Therapy for Acute Myeloid Leukemia in Child, Adolescent, and Early Young Adult Patients: The MD Anderson Cancer Center Experience
Source: Cancers (Basel). 2024 Nov 20;16(22):3886. doi: 10.3390/cancers16223886 (PMC11592422; doi:10.3390/cancers16223886)
Supplement: Supplementary file 1 [file cancers-16-03886-s001.zip › Supplemental Table S1_updated_clean.pdf]

Supplemental Table 1: Treatment Details of Cladribine-Containing Regimens				
**Regimen**	**Dosing Details**			**Response Rate on this Study (CR, CRi)**
CLIA	Cladribine<br>Idarubicin<br>Cytarabine	5 mg/m^2/dose daily<br>10 mg/m^2/dose daily<br>1.5 mg/m^2/dose daily	Day 1-5<br>Day 1-3<br>Day 1-5	40%
CLIA + Gilteritinib	Cladribine<br>Idarubicin<br>Cytarabine<br>Gilteritinib	5 mg/m^2/dose daily<br>10 mg/m^2/dose daily<br>1.5 mg/m^2/dose daily<br>120 mg daily	Day 1-5<br>Day 1-3<br>Day 1-5<br>Day 1-14	N/A*
CLIA + Midostaurin	Cladribine<br>Idarubicin<br>Cytarabine<br>Midostaurin	5 mg/m^2/dose daily<br>10 mg/m^2/dose daily<br>2 mg/m^2/dose daily<br>25 mg every 12 hours	Day 1-5<br>Day 1-3<br>Day 1-5<br>Day 2-4	0%
CLIA + Quizartinib	Cladribine<br>Idarubicin<br>Cytarabine<br>Quizartinib	5 mg/m^2/dose daily<br>10 mg/m^2/dose daily<br>1.5 mg/m^2/dose daily<br>20 mg daily	Day 1-5<br>Day 1-3<br>Day 1-5<br>Day 6-19	N/A*
CLIA/Venetoclax	Cladribine<br>Idarubicin<br>Cytarabine<br>Venetoclax	5 mg/m^2/dose daily<br>10 mg/m^2/dose daily<br>1.5 mg/m^2/dose daily<br>7, 14, or 21 days at 400 mg or 235 mg/m^2	Day 1-5<br>Day 1-3<br>Day 1-5<br>Day 2-	55%
CLIA/Venetoclax + Gilteritinib	Cladribine<br>Idarubicin<br>Cytarabine<br>Venetoclax<br>Gilteritinib	5 mg/m^2/dose daily<br>10 mg/m^2/dose daily<br>1.5 mg/m^2/dose daily<br>7, 14, or 21 days at 400 mg or 235 mg/m^2<br>120 mg daily	Day 1-5<br>Day 1-3<br>Day 1-5<br>Day 2-<br>Day 1-14	50%
CLIA/Venetoclax + GO	Cladribine<br>Idarubicin<br>Cytarabine<br>Venetoclax<br>Gemtuzumab ozogamicin	5 mg/m^2/dose daily<br>10 mg/m^2/dose daily<br>1.5 mg/m^2/dose daily<br>7 or 14 days at 400 mg or 235 mg/m^2<br>4.5 mg or 3 mg/m^2 each day	Day 1-5<br>Day 1-3<br>Day 1-5<br>Day 2-<br>Day 1 OR Day 1 and Day 4	67%
CLAD/ARAC + Venetoclax	Cladribine<br>Cytarabine<br>Venetoclax	5 mg/m^2/dose daily<br>1.5 mg/m^2/dose daily<br>400 mg max or 235 mg/m^2	Day 1-5<br>Day 1-4<br>Day 2-8	33%

CLAD/LDAC + Venetoclax	Cladribine<br>Cytarabine<br>Venetoclax	5 mg/m^2/dose daily<br>75 mg/m^2/dose twice daily<br>400 mg or 235 mg/m^2	Day 1-5<br>Day 1-10<br>Day 1-21	
CLAD/ARAC	Cladribine<br>Cytarabine	9 mg/m^2 daily<br>500 mg/m^2/day continuous IV infusion	Day 1-5<br>Day 1-5	50%
CLAD/ARAC + GO	Cladribine<br>Cytarabine<br>Gemtuzumab ozogamicin	5 mg/m^2/dose daily<br>2 mg/m^2/dose daily<br>4.5 mg or 3 mg/m^2/dose	Day 1-5<br>Day 1-5<br>Day 1	33%
Abbreviations: ARAC, cytarabine; CLAD, cladribine; CLIA, cladribine, idarubicin, cytarabine; GO, gemtuzumab ozogamicin; LDAC, low-dose cytarabine. *These two combinations were used once on this study when patient was already in remission				
